# Compassion-Focused Technologies: Reflections and Future Directions

**DOI:** 10.3389/fpsyg.2021.603618

**Published:** 2021-05-13

**Authors:** Jamin Day, Joel C. Finkelstein, Brent A. Field, Benjamin Matthews, James N. Kirby, James R. Doty

**Affiliations:** ^1^College of Health, Medicine and Wellbeing, Family Action Center, The University of Newcastle, Newcastle, NSW, Australia; ^2^School of Medicine, The Center for Compassion and Altruism Research and Education (CCARE), Stanford University, Stanford, CA, United States; ^3^Princeton Neuroscience Institute, Princeton University, Princeton, NJ, United States; ^4^School of Creative Industries, College of Human and Social Futures, The University of Newcastle, Newcastle, NSW, Australia; ^5^School of Psychology, Faculty of Health and Behavioral Sciences, The University of Queensland, Brisbane, QLD, Australia

**Keywords:** compassion, digital technology, perspective, extended reality, human society, social media

## Abstract

Compassion is a prosocial motivation that is critical to the development and survival of the human species. Cultivating compassion involves developing deep wisdom, insight, and understanding into the nature and causes of human suffering; and wisdom and commitment to take positive action to alleviate suffering. This perspective piece discusses how compassion relates to the context of modern technology, which has developed at a rapid pace in recent decades. While advances in digital technology build on humankind’s vast capacity to develop practical tools that promise to enrich our lives and improve our social connections, in reality the effects are often far from benign. The motives underlying the development of many contemporary digital platforms seem rooted in competitiveness and capitalism; while modern social media and online platforms are having a profound and pervasive impact on the mental health and wellbeing of humans around the globe. Nonetheless, digital technology holds considerable potential to promote compassionate insight, wisdom, and prosocial behavior. We reflect on the current state of technology within human society and examine the notion of *compassionate technologies*; discuss how contemporary paradigm shifts such as the inclusive design movement may be harnessed to build tools and platforms that promote collective good and increase prosocial behavior; and highlight examples of initiatives that are harnessing modern technology to advance democracy, collective knowledge, and personal freedoms and agency.

## Introduction

According to the Dalai Lama, “Love and compassion are necessities, not luxuries. Without them, humanity cannot survive” (as cited in Powys, 2011). In recent decades, compassion researchers have increasingly recognized that compassion is not merely an ideological virtue, but a complex feature of our socio-biology that is critical to our survival as a species. Compassion is motivated by powerful neural pathways that are stimulated by suffering, with corresponding neurological reward centers stimulated by alleviating suffering. When compassion is cultivated, the mind takes on characteristics of openness, courage and understanding (Gilbert, 2014).

With few exceptions, a central tenant of the evolution of complex biological systems has been one of cooperation. This has never been more evident than in the human species where there is an absolute requirement that our offspring are nurtured, cared for and protected for well over a decade before they are capable of functioning independently. To ensure that such effort is expended in terms of energy and resources, a complex reward system evolved in our nervous systems. Compassionate behavior stimulates areas of the brain associated with pleasure and carries profoundly positive effects in our peripheral physiology (Goetz et al., 2010). During humanity’s hunter-gatherer phase, the capacity to recognize emotional states in others fostered greater nurturance, cooperation, regulation and collective defense (Gilbert, 2019). Compassion, the fundamental ability to recognize suffering and be motivated to alleviate it, has been demonstrated to be necessary for the long-term survival of human life (Williams, 2018).

In this paper, we discuss the future of compassion in relation to technology. A critical question for the current age is: Can technology be used to further our scientific understanding of compassion; and to steer us toward greater personal autonomy, collective agency, empathy, and compassionate action? In the following sections, we discuss how modern technology has been a defining feature of the 21st Century, shaping humanity at multiple levels including the social, behavioral, cultural, and ethical. Technological proliferation has increased connectivity, lowered global barriers, and provided new avenues for trade and livelihood; while also fomenting greed, competitiveness and comparative individuals and societies. We contend that compassionate technologies are those that harness contemporary advances to actively restore, humanize and strengthen our relationships with one another as well as ourselves, and discuss examples of new mechanisms, modalities and emerging fields that support these aims.

### The Role of Technology in the 21st Century

Technology has long played a significant role in the evolution of the human species. In the 21st Century, we have witnessed an explosion of data and digitization. The pace of technological evolutions has occurred rapidly, by orders of magnitude and with seeming exponential growth. From advances in nanotechnology, bioengineering, neuroscience, information technology, artificial intelligence and intelligence augmentation, to 3D printing and virtual reality, technology continues to permeate every part of our society. Broadly speaking there are few signs that thoughtful care and consideration has been given to the socio-technical implications of this growth, at least not to a degree that is commensurate with the rapid pace of technological development. Between financial firms, social networks and digital vendors, we live in the shadow of a computational arms race to obtain, analyze, and model as much information as possible, with increasing impacts to our daily lives. As technology has gradually shifted from production and industrial output to the organizing of information and complex social systems, a complex web of interconnected services has emerged that encompasses a wide range of domains: from search to social life, finance and digital health. The majority of citizens in Western society now regularly straddle between digital and “analog” lives, usually with little knowledge of the degree of behind-the-scenes information gathering that takes place in the digital sphere regarding our collective behavior, friendships, spending, and interests.

The networks that power these daily interactions tend to abstract resources and information away from the public eye for commercial or political benefit, rather than making those resources (i.e., data and information) openly accessible and available. Across many areas, this has fostered an underlying winner-takes-all zeitgeist that confers increasing advantages as scale increases. Nick Srnicek (2017) has described this as a shift toward “platform capitalism,” allowing those platforms with the largest user-bases to provide a foundation from which others emerge and operate across a range of sectors. By virtue of this, a defining characteristic of the current era is that organizations with the greatest informational and computational resources exhibit growing dominance; while these in turn contend for an ever-increasing number of users in a bid to influence and shape consumer behavior.

These digital networks are having a profound effect on our actions and decision making (Lessig, 1999). Our daily activity, financial spending, browsing history, and social lives have each succumbed to a pervasive new culture of technological voyeurism and influence. Similarly, modern luxuries including GPS, wearables and smart-home devices acutely study users’ daily habits. Via these mechanisms, governments and commercial industries have increasingly sought to map out society through statistical representations and “big data,” embracing large-scale infrastructure and machine learning algorithms that can predict personal consuming habits and classify social identities, habits and preferences. This type of modeling has become a lynchpin of modern commercialism. Even brick and mortar stores increasingly rely on Wi-Fi signals and sophisticated video surveillance offerings to track individual customer’s dwell time, gender, mood, return rate, and other personal information. Networks progressively link data on the user’s behavior with other personal information that can be obtained relatively cheaply from companies that specialize in data aggregation. Through expanding databases of personal information, predictive analytics have already improved in accuracy enough to precisely assign an individual’s age, gender, financial status, purchasing habits, political opinions, as well as many other dimensions of their personality and habits. They then aim, with laser precision, to deliver custom tailored incentive offerings, advertisements and suggested purchases at the exact times and places that such feedback will maximize impact in a decision process (see Sadowski, 2020 for a more in-depth discussion).

In the information age – what Stiegler (2019) has called “the age of disruption” – and the era of social media, the impacts of constant technology use have grown more pervasive. Virtual networks profoundly influence our perception of time (Kweon et al., 2011), and give a sense of simultaneous connectedness and instantaneous feedback that make it difficult to distance ourselves from the distraction of the digital sphere (Kraushaar and Novak, 2010; Rosen et al., 2013). As Lanier (2014) argues, under this arrangement we tend to forfeit what we would thoughtfully wish for ourselves, to gain distraction through personalized, interactive desires. Our decisions become constrained by both herd-mentality and a bevy of ranking and clustering algorithms that model our choices before we make them. Furthermore, the richness and reciprocity available to us during offline interactions can become compressed by virtual networks down to a unidimensional portrayal of society and the individuals that occupy it. Relative to digital space, the affective dimension of in-person, embodied experience of others provides a very different opportunity to model and test our world making and decisions through intimate communication with trusted others. The full impact of a diminution of such offline interaction is beyond the scope of the current discussion, but certainly includes a flattening of not just our independent capacity to engage in decision making, but also our access to the sociality of direct human interaction where these decisions can be tested against the subjective experience of others.

Each day, individuals communicate instantaneously and are inundated by unprecedented floods of digital information, yielding what Fries (2012) describes as “information asymmetry”; overwhelming access to information, the veracity and implications of which most cannot discern. Indeed, the emerging consensus has been that the time spent in virtual interactions is beginning to eclipse face-to-face communication (Bureau of Labor Statistics, 2014). All this, while staring down existential threats of virus pandemics, nuclear or mechanized warfare, and environmental catastrophes. Recently, with worldwide events including the global COVID-19 pandemic, these widespread sociological shifts have become even more entrenched within modern society.

Social networks such as Facebook, Instagram, Twitter and others have heralded a new age of connectivity, with a two-way flow of information that bridges historical barriers associated with class, country, race and status. Individuals now have direct access to opinions, insights and images from thought leaders, world leaders, and celebrities. The dispersion of information undoubtedly carries some putative benefits for producing more compassionate societies. For example, first-hand access to information from those embedded in war zones, or in countries ravaged by famine or social injustice, alongside the promotion of online campaigns and petitions, may prompt greater individual care, awareness and compassionate action than would have been previously possible through traditional media only. Yet concerningly, in Western society at least we have witnessed an alarming reduction in humanity’s capacity to understand and empathize with ourselves and one another (Konrath et al., 2011; Twenge et al., 2012); a trend that worryingly corresponds to the growth of virtual social networks and smartphones. This poses a significant threat to the collective wellbeing of modern societies, and promises to continue alienating and fragmenting our broader social selves. The experience of virtual sociality, depending on its use, has proven fertile ground for habitual narcissism (Gentile et al., 2012; Horton et al., 2014), with our worst indulgences often decoupled from immediate consequences and feedback (Turkle, 2011). It is disconcerting that this relative anonymity seemingly mutes many of the perceived consequences of violent or aggressive social communication, birthing an epidemic of cyberbullying and hostile “trolling” (Villines, 2015). Indeed, literature shows a correlational link between excessive use of virtual sociality and a cavalcade of negative psychological outcomes, including loss of well-being (Kross et al., 2013), poor relationship outcomes (Morgan et al., 2016), such as cheating, divorce and breakups (Clayton et al., 2013), increases in narcissistic aggression (Carpenter, 2012), poor academic performance (Junco, 2015), and envy (Verduyn et al., 2015).

Looking forward, major research programs from industry and scholarly settings are increasingly emphasizing virtual sociality as playing a vital role within our daily lives and routines. As wearables and mobile technologies grow more sophisticated, this raises complex ethical challenges. For instance, an “AR Cloud,” which is in the simplest sense a contemporaneous digital copy of the world most commonly implemented via augmented reality (AR), has become the latest mechanism by which connected services gain personalized access to users’ lives and data. Meanwhile, the European Commission funded VRTogether project and Facebook’s Reality Labs have developed photo-realistic 3D avatars for use in virtual reality (VR) conferencing and other shared social experiences (De Simone et al., 2019; Gunkel et al., 2019; Li et al., 2019; Rubin, 2019; VRTogether, 2020). Facebook call these “Codec Avatars”: photorealistic computer-generated avatars based on high resolution image gathering and machine learning that builds a model of how our clothing and bodies move that can react dynamically to the sound of a voice (Rubin, 2019). The high fidelity of these technological advances holds exciting potential, presenting new opportunities for the future of remote work, social connectedness, and even psychotherapy (e.g., Bertrand et al., 2018; Kothgassner et al., 2019). Conversely, left unchecked, the potential for misuse is great, exposing new mechanisms for dangers such as identity theft or trauma for those individuals who relate deeply through immersive virtual experiences.

### Compassionate Technologies

We acknowledge this synopsis of the current state of technology sounds downcast, perhaps even ominous. Of course, modern advances in digital technology offer far more positives than we have alluded to here, including universal, real-time access to global information; new labor market opportunities; meaningful and creative contributions to digital and actual society; and platforms that foster connection and community. Nonetheless, we harken back to one of the central tenets of compassion, defined as “the sensitivity to suffering in self and others, with a commitment to try to alleviate and prevent it” (Gilbert, 2014). In other words, before we can identify and commit to a way forward that seeks to prevents human suffering, we must demonstrate wisdom and insight to *recognize and understand* the cause of suffering (Sternberg, 2012)—and the role of technology in preventing, alleviating or exacerbating it. Clearly, technology has the potential to engender global threats and collectively deteriorate compassionate and humanistic thinking. Fortunately, technological evolution, unlike biological evolution, is not dictated merely by circumstance. The values we wish to engender and the human identity we wish to foster, can influence technological evolution itself. We argue that technology has significant potential to foster collective agency, empathy, and mutual understanding. The crucial distinction is one of design, intent, and the repercussions of design choices. The culture of innovation we create now will determine which of these contending eventualities will prevail.

An example of this in action can be found in the principles of inclusive design. People having difficulty accessing and/or using products and services are frequently excluded from consideration in design processes with the effect of limiting the design output’s efficacy, versatility, lifespan, and commercial potential (Amin, 2019). Inclusive design approaches, alternatively, consider diverse users in an effort to drive innovation and improve experiences for all participants. Here, inclusive design is defined as “a human centered or user centered design methodology that provides a framework to understand the needs, wants, and limitations of end users” (Amin, 2019). The goal is to increase both the human and commercial potential of a product or service. From a human centered perspective, therefore, we might frame inclusive design as an ethical approach that considers the needs, wants, and limitations of end users within various contexts as separate and unique challenges, with an aim to deliver discrete solutions that demand consideration of diverse perspectives. The argument on behalf of an inclusive approach to design, therefore, not only promotes greater access; it also attends to the quality and implications of that access for all.

There are now critical opportunities for new technologies within a space we nominally refer to as “compassionate technologies.” Compassionate technologies seek to restore and humanize our relationships with one another, and to our sense of self. We suggest that compassionate technologies might achieve this through two key aims. The first key aim for these technologies is *to democratically reconstitute access and control of digital and physical networks through platform cooperativism so as to foster greater freedom and compassion in groups.* We define platform cooperativism to be the collective ownership of and citizenship in digital platforms. These platforms serve to share resources cooperatively, sustainably, and automatically. They can democratically facilitate a wide range of community and social functions, such as ridesharing and room sharing, to energy sharing and neighborhood security.

These platforms are not to be confused with the so-called “sharing economy.” Companies such as Uber for ridesharing, Spotify for music, and Airbnb for room sharing, have the capacity for similar resource distribution and cooperation. But, as Scholz points out, these hierarchical organizations centrally commodify the act of sharing so that the company becomes its chief financial beneficiaries (Scholz, 2016). The sharing economy flouts labor protections and asymmetrically extracts benefits, profit and information from the network.

Platform cooperatives instead seek to decentralize ownership, democratize access to information, and protect the benefits of its workers and content producers alongside its users. There are already examples of such platforms around the world that have demonstrated significant social benefits. For example, *prod-user* owned platforms, wherein producers co-own the platforms to which they are selling their work, yield careers and returns for their content producers. Examples include platforms for photographers (*Stocksy*)^1^, filmmakers (The Film-Makers’ Cooperative)^2^ and musicians (*Resonate*)^3^. Additionally, cooperative online marketplaces are emerging, such as *Fairmondo^4^*, a fast-growing, user-owned service similar to Ebay, which has taken hold in Germany. Other platform coops exist such as the *Loconomics* movement^5^, a community-owned cooperative labor brokerage offering services from babysitting to car-repair^6^. In the context of modern neoliberal capitalist societies, decentralized platforms such as these are not immune from challenges. Ensuring long-term viability depends on sufficient economic returns and the capacity to grow the user base to a critical mass that affords sustainability and attracts content creators or distributors; while other potential pitfalls such as disentangling intellectual property rights or balancing commercial interests with user interests must be navigated (e.g., Bruns, 2007).

Experiments in more human-centric, agile, and distributed political governance also promise to re-define and expand the adaptability of governance. Experimental governmental organizations such as the United Kingdom’s Nudge Unit (Behavioural Insights, 2016) and Denmark’s Mind Lab (2016) promote policy based on “open city” and big data stores in combination with corresponding behavioral economics. These groups use data-intensive methods. They couple these to insights into human behavior to construct a more benign society. Others, such as Finland’s Design for Government Initiative (Annala et al., 2015), take the process of iterative design further by encouraging massively cooperative feedback from citizens in experimental policy trials. Here, citizens are participants in both data collection and suggestions for new hypotheses. Meanwhile, new initiatives focusing on massively distributed governance, or “deep democracy,” provide new opportunities for technological experiments in public and participatory decision-making. For example, *Liquid Democracy* ruptures the historical ties of governance to the elite and seeks to overcome impediments to community engagement in decision-making (Ramos, 2015). This cloud platform permits groups or organizations to collectively deliberate and decide on relevant issues pertaining to their common purpose. “Liquid Feedback” (Behrens et al., 2014) combines both representative and direct deliberation systems and allows any user to act as both political representative and voter. Members can deliver their own voting authority as a proxy vote to one another, supplanting the need for career politicians. They can delineate these proxies to specific members. They can constrain that mandate to explicit policy categories and for specific time spans. These proxies are highly flexible and rescindable. They permit a “liquid” flow of power through a network where anyone can make a proposition, have an exchange, engage in deliberation or delegate authority rapidly.

This type of human-centric governance has potential to achieve policy and design breakthroughs through deep collaboration with local citizenry. Participatory technologies could empower our collective stewardship of our environments through sharing of knowledge resources and assisting services (Ahn et al., 2004). Enabling access to diverse views and opinions is useful where conflict, negotiation and compromise must ensue to share resources or make collective decisions. Through distributed collaboration, access to open data and civic records, capacity for instantaneous feedback, and collectivized decision making, these new forms of compassionate technologies can make empathetic concern for one another the basis of their functioning. The data they generate becomes a possession of the communities they serve: a data store of our collective needs. However, such self-governance requires a culture of civic engagement. For an organization or community to exercise a sense of self and a commitment to shared courage and compassion, a shared sense of self needs to emerge with common ideals, ethics and values.

A second critical aim of compassionate technologies is *to use technology to advance, rather than hinder, cognitive liberty; to foster greater freedom and compassion in relation to ourselves.* There are two chief ways that technology can aid the preservation of agency. First, technology can help us better understand and regulate our moment-by-moment emotions, thoughts, and feelings. It can help us create a more textured and compassionate representation of our own needs and the needs of others. Second, technology can help us defend our “soulfulness,” agency and considered goals against distraction and mindlessness. Compassionate technology must also seek to transform the dilemma of surveillance and control into an empathetic fixture for greater compassion and awareness. As Harris (2016), argues the hidden, algorithmic influences that subvert our agency often do so by modeling our impulses. They then confine our attention to a menu of highly personalized, and often distracting options. What if we could instead imbue our environment with values that promote what we “want to want” for ourselves? Here, the same tools that provide influential capabilities to marketers may be subverted into tools for self-reflection and compassion in a closed loop – facilitating a greater capacity to understand ourselves. Virtual assistants in this scenario may incorporate a mindful and compassionate context, to algorithmically steer users toward motivated empathy, solitude and reflection through emotional analytics that provide deep insights into our state of mind on a moment-by-moment basis. For example, these tools may reinforce how our tone of voice, our posture and word choice are reaching others and how others, in turn, are affecting us.

Finally, development in compassionate technology must seek to imbue virtue within virtual interactions. How can we develop digital formats that better engender heartfulness and pro-social behavior? Pro-social cues and contexts in virtual interaction are now being studied to see how they might enhance compassion. This is especially relevant for virtual reality, which draws on innate heuristics such as presence, closeness and social connection while instantaneously bridging time and space in ways that have previously been out of reach, opening up new opportunities for individuals to engage with experiences that build empathy, compassion and prosociality (e.g., Bertrand et al., 2018). For example, contemporary practices such as loving-kindness and compassion meditations are increasingly being recognized for their benefits through both clinical research as well as brain imaging and neuroendocrine studies (Hofmann et al., 2011). Typically, individuals engage with these activities on a personal level, or perhaps in a clinical setting with guidance from a therapist. With recent advances in technology, numerous, untapped possibilities now abound in this space, including novel mechanisms for integrating therapeutic activities within socially connected, digital spaces; though little is yet known about the potential benefits—or drawbacks—from utilizing such practices in this way.

### Conclusion

A wealth of evidence suggests we can enhance the likelihood of compassionate states and prosocial behavior via modern digital technology. Armed with advances in the science and technology of compassion, the acceleration of technological evolution may be steered to achieve a greater emphasis on positive feedback cycles of trust, reciprocal interaction and self-discovery. In the face of the explosive growth of technological power in the 21st century, compassion calls on us to courageously revisit our social contract. A new vista has opened in technological design which, for the first time, permits us to participate more fully and immediately in our own lives and those of others. The courage to participate and engage with our conditions, and the new technologies that catalyze this effort, together portend the possibility of a new social contract for the 21st century – enacted reciprocally, with one another and for mutual benefit. To this end, significant legal and economic reform, “venture cooperatives” for financing, and willing communities will be needed. As will a design philosophy that is human centered, and oriented by inclusivity. Most of all, it requires advocates for these reforms who recognize the centrality of compassion to the historical arc of human evolution and, ultimately its requirement for our survival.

## Data Availability Statement

The original contributions presented in the study are included in the article/supplementary material, further inquiries can be directed to the corresponding author.

## Author Contributions

All authors contributed to the drafting, editing, and reviewing of this manuscript and approved the submitted version.

## Conflict of Interest

The authors declare that the research was conducted in the absence of any commercial or financial relationships that could be construed as a potential conflict of interest.

## Footnotes

1 https://www.stocksy.com/

2 https://film-makerscoop.com/

3 https://resonate.is/

4 https://www.fairmondo.de/global

5 http://coop.loconomics.com.au/

6 https://platform.coop/
